# Successful treatment of late Salmonella infections in total hip replacement - report of two cases

**DOI:** 10.1186/1471-2334-10-160

**Published:** 2010-06-08

**Authors:** Kálmán Tóth, Gábor Janositz, Gyula Kovács, Krisztián Sisák, Ervin Rudner

**Affiliations:** 1 Department of Orthopaedics, University of Szeged, 6 Semmelweis Street, Szeged, 6720, Hungary; 2 Department of Orthopaedics, County Hospital of Kecskemét, 39 Nyíri Road, Kecskemét, 6000, Hungary; 3 Department of Orthopaedics, County Hospital of Szolnok, 21 Tószegi Street, Szolnok, 5004, Hungary

## Abstract

**Background:**

Salmonella species can be rarely isolated from periprosthetic joint infections, however when present, are usually part of a severe septic clinical picture.

**Case presentations:**

Two patients presented with late infected hip replacements to our institution. The first patient with multiple comorbidities had a confirmed Salmonella Enteridis infection with an abscess in the groin, with loosening of both components. He underwent a successful one stage cemented revision hip replacement, followed by 6 weeks of antibiotic therapy (ciprofloxacin). He had no recurrence or complications. The second patient was admitted in a septic condition with ARDS to the Intensive Care Unit 7 years following an uncemented total hip replacement. From an ultrasound guided hip aspirate Salmonella cholerae-suis was isolated. He underwent a successful a two-stage revision hip replacement.

**Conclusions:**

Successful treatment of such potentially life threatening infections is achievable using modern orthopaedic techniques and close collaboration with the infectious diseases specialists.

## Background

Septic arthritis is a rare complication of salmonella bacteraemia. In a large series of bacterial isolates, Salmonella species could be isolated from approximately 1.6% of the joints [1]. Several Salmonella species have been reported to cause periprosthetic joint infection. Salmonella typhi murium and Salmonella enteritidis were found to be the most common serotypes in bone infections, whereas members of the C1 serogroup were the most common cause of septic joint infections [2]. There have only been a very few cases of infections due to Salmonella cholerae suis, which is epidemiologically an extremely uncommon serotype (0,01%) [2].

## Case presentations

A 77-year-old man presented to the outpatient department with hip pain after falling. Two years earlier, he underwent an uncomplicated primary hip replacement (MY cup and stem, Protetim Ltd., Hungary). Initial radiographs were negative, and the patient was discharged, with the advice of rest and a further follow-up. 6 months later, he presented again, with constant pain in his right hip and had a four day history of high temperature and a swollen lump in the inguinal area, apparently an abscess. The aspiration of the abscess grew Salmonella Enteridis. Laboratory findings included a positive salmonella O antibody titer (1:400), a very high erythrocyte sedimentation rate (120 mms/h), hypalbuminaemia and anaemia. Radiographs showed osteomyelitis and periosteal reaction around both components, in the acetabulum and in the femur.

Because of the patient's advanced age and poor medical condition, before proceeding with treatment of the infection, the patient needed a temporary pacemaker. Although a two-stage revision would have been the ideal procedure for the patient, considering his cardiovascular status, upon consulting with the anaesthetic team, a one-stage procedure was chosen to decrease perioperative stress, and to provide an immediately weight bearing limb, with earlier mobility. From an anterolateral approach, the abscess was drained, the walls of the abscess were excised, and a cemented cup and cemented revision stem (MY cup and stem, Protetim Ltd., Hungary) were implanted in one sitting. The patient was started on intravenous antibiotics according to the susceptibility pattern of the microorganism. The Kirby Bauer disc diffusion test was used for susceptibility testing (non-automated). In the initial 72 hrs 3 × 400 mgs of ciprofloxacin was given, which was switched to oral ciprofloxacin for a further six weeks, in the dose of 2 × 500 mgs. Although the organism was not sensitive to gentamicin we used our routine gentamicin containing bone cement to fix both components, as this was the only antibiotic loaden bone cement available to us at that time. By the second postoperative day the patient had a normal temperature and a subsequent haemoculture, urine samples and a wound swab were all negative. The patient was discharged on the 13^th ^day. The Salmonella antibody titer was down to 1:100. The patient was symptom and complication free for a further 6 and half years, and died of unrelated causes at the age of 84.

A 61-year-old male patient had bilateral uncemented hip replacements (MY uncemented cup and stem, Protetim Ltd., Hungary) because of avascular necrosis of the both femoral heads. A year later the right hip was revised because of a fracture of the ceramic head. For the next six years the patient was asymptomatic. Seven years after initial surgery, he presented with dislocation of the right hip prosthesis which occurred after a fall. The hip was reduced and the patient was discharged three days later. One day after discharge he was admitted to a medical ward with tachypnea, atrial fibrillation and fluid in the lungs. Laboratory findings included normal anion gap, elevated chlorine level, metabolic acidosis, deteriorated kidney and liver functions, jaundice and severe thrombocytopenia. Despite receiving oxygen, his oxygen saturation did not rise above 95% indicating potential parenchymal lung damage. Massive pulmonary embolism was clinically excluded. The working diagnosis was of ARDS caused by sepsis. The patient was transferred to the intensive care unit and was intubated because of progressive respiratory insufficiency. In the medical ward an empirical combination of intravenous antibiotics was initiated, which included cefotaxime 3 × 2 grs, supplemented by metronidazole 3 × 500 mgs daily as the initial working diagnosis was gastrointestinal infection with jaundice. Because of the imminent multiple organ failure the antibiotic treatment was changed in the intensive care unit to 3 × 4.5 grs of piperacillin.

After some improvement in his general condition a more thorough search for the cause of infection was carried out including an autolog leukocyte scintigraphy. The results showed increased uptake in both lungs, in the spleen, and in the soft tissues around the right hip. His general condition did not allow any surgical intervention at that stage. During the next ten days several haemoculture samples were taken and they were all negative. The only positive finding was a pharynx swab showing Candida albicans, which was felt to have no clinical significance. Then an ultrasound guided aspiration of the right hip was performed, gaining sufficient samples for microbiology. Samples showed a mixed flora of Salmonella cholerae-suis, and Acinetobacter haemolyticus.

As per the susceptibility of the organism intensive, intravenous, combined antibiotic treatment of 3 × 600 mgs of clindamycin and 3 × 400 mgs of ciprofloxacin was initiated for the first 72 hrs, and oral ciprofloxacin was given for a further 6 weeks, in the dose of 2 × 500 mgs. The patient's condition improved enough to perform a revision of the right total hip replacement. Both components were found to be stable, however they were removed along with a thorough debridement of the abscess. For the removal of the stable stem, it was necessary to open a cortical window on the femur. This was fixed with three cerclage wires. A spacer containing Gentamicin was inserted into the femur and the acetabulum. The intra-operative cultures, a haemoculture sample and the surgical drain were all returned negative for microorganisms.

C reactive protein and ESR values returned to the physiological threshold after three months. However, there was a further month of delay before the second stage reimplantation could be performed because of extensive sacral and calcaneal pressure sores suffered in the initial three weeks of intensive care.

An uncemented revision hip replacement was performed using a Duraloc cup and AML stem (DePuy, Warsaw, In.). Just four months after the reimplantation, the patient went back to his original occupation and at latest follow-up, five years post-operatively he is asymptomatic and there has been no sign of recurrent infection.

## Conclusions

Septic complications after total hip replacement are infrequent but serious complications of total hip replacement.

Several studies have reported that debridement with retention of the original components might be less successful than removing the infected joint replacement [3-5]. However, debridement and retention can be successful providing that an antibiotic with efficacy on biofilm bacteria (surface-adhering bacteria) is used [6]. This strategy might be effective for patients unfit for more extensive surgery.

In the English literature, there have only been 10 reported cases of post-operative infections caused by Salmonella species after total hip replacement [7-12]. Salmonella species have been associated with pediatric septic arthritis and there have been recent reports of septic arthritis of the hip caused by Salmonella typhi in children [13,14]. Out of the 10 cases of periprosthetic infections four were early infections ([11], and [12] case No.1,2,3) and in two of these four the implant could be retained with prompt surgical debridement and appropriate antibiotic therapy ([11], and [12] case No.3). In one of these cases a recurrence of the infection developed 18 months after the debridement, but with the use of constant oral antibiotic therapy, this was suppressed [11]. Out of the six late infections published ([7-12] case No.4), one-stage replacement was described in only one case [7], who was unsuccessfully pretreated with co-trimoxazole for 16 months before implant-replacement. In the other 5 cases, two-stage exchange was performed ([8-12] case No.4).

In our first case, due to the patient's advanced age and poor cardiovascular status, we felt that he would not be a good candidate for a two-stage revision involving two potentially long operations and a long rehabilitation period in between, which involves no weight bearing on the affected limb. After considering both options, a single stage revision was performed. This was successful in eradicating the infection and providing a functional hip joint early. One stage revisions are not usually mentioned in treating infectious complications following hip replacement caused by Salmonella species, but is a surgical option used in orthopaedic surgery to treat infectious complications caused by other microorganisms.

In our second case, an extremely rare and quite aggressive serotype of Salmonella caused the infection resulting in sepsis requiring intensive care and resulting in multi-organ failure. In the current English literature we have been unable to find any cases, where reimplantation of a hip replacement was possible under such difficult circumstances. There has been only a single one-stage replacement in the literature, after Salmonella infection, but in that case, this was preceded by more than a year-long antibiotic treatment [7]. Our patient returned to his original occupation and activity level 8 months after the first stage of the two-stage procedure and only 4 months after the reimplantation. Using an uncemented implant during reimplantation in such an infection has not been previously published.

Despite the small number of cases, our experiences can demonstrate that even in late haematogenous infections, caused by rare microorganisms, such as the two cases described, using otherwise well defined guidelines and procedures available to modern orthopaedics for the treatment of infectious complications, it is possible to achieve fast recovery and successful long term results without risking further recurrence. The fast and meticulous eradication of the infection is paramount as such serious infections can be life threatening. The advantages and disadvantages of available treatment options have to be weighed, with emphasis on the patients' medical status. Not confining treatment to established patterns can result in quick and effective treatment even in very serious infections such as Salmonella infections.

## Abbreviations

ARDS: Acute Respiratory Distress Syndrome;

## Competing interests

The authors declare that they have no competing interests.

## Authors' contributions

All authors contributed to this work. GJ, GYK were involved in the direct clinical care (diagnosis, decision making, treatment, operative intervention, postoperative care) of patient 1. ER was involved in the direct clinical care (diagnosis, decision making, treatment, operative intervention, postoperative care) of patient 2. KT and KS were involved in the direct clinical care of both patients. All authors read and approved the final manuscript.

## Pre-publication history

The pre-publication history for this paper can be accessed here:

http://www.biomedcentral.com/1471-2334/10/160/prepub
